# Influence of climate factors on pediatric alopecia areata flares in Philadelphia, Pennsylvania

**DOI:** 10.1038/s41598-021-00433-0

**Published:** 2021-10-26

**Authors:** Elisabeth A. George, Leslie Castelo-Soccio, Elana Putterman, Helena Kuhn, Carlos Wambier, Abrar Qureshi, Eunyoung Cho

**Affiliations:** 1 Warren Alpert Medical School of Brown University, Providence, RI USA; 2 Division of Pediatrics, Section of Dermatology, The Children’s Hospital of Philadelphia, Philadelphia, PA USA; 3 University of Pennsylvania Perelman School of Medicine, The University of Pennsylvania, Philadelphia, USA; 4 Department of Dermatology, Warren Alpert Medical School of Brown University, 339 Eddy Street, Providence, RI 02903 USA

**Keywords:** Climate sciences, Paediatric research

## Abstract

Patients with alopecia areata (AA) may experience episodic disease flares characterized by increasing hair loss that follow a seasonal pattern. However, no studies have examined whether specific climate factors contribute to the seasonal pattern of AA flares. Using Spearman rank correlation analyses, we assessed the association between climate variables and AA flare frequency per month in 336 children with AA in Philadelphia, Pennsylvania. Region-specific monthly values for average ambient temperature, air pressure, cloudiness, hours of sunlight, relative humidity, number of days with sun, number of days with rain, volume of precipitation, wind gust, wind speed, and UV index from January 2015 to December 2017 were obtained from World Weather Online. We found significant (P < 0.05) correlations between AA flare frequency and UV index (R = − 0.66), precipitation (R = − 0.66), number of days with rain (R = − 0.70), number of days with sun (R = 0.62), and air pressure (R = 0.80). Stratified analyses showed even stronger associations with UV index and precipitation in patients with an atopic comorbidity. New significant correlations appeared with temperature, wind speed, and UV index of the prior month. However, in patients who did not have atopic comorbidities, we generally observed weaker and non-significant correlations between climate and AA flare frequency. This study suggests that certain climate factors may mediate the seasonal pattern of AA flares and may contribute to AA pathogenesis. Atopic AA patients may be more susceptible to the influence of climate compared to those with no history of atopy.

## Introduction

We previously found that pediatric alopecia areata (AA) flares follow a seasonal pattern, with a greater proportion of flares occurring in the late fall and a smaller proportion towards the end of spring^1^. Stratified analyses based on the presence of an atopic comorbidity suggest that the clinical course of AA differs between atopic patients and their counterparts^1^. We found that the greatest proportion of flares occurred during the winter in atopic AA patients. However, the greatest proportion of flares occurred during the fall in patients without atopy.

Whether climate factors contribute to the seasonal pattern of pediatric AA flares remains unknown. In the present study, we hypothesize that regional UV index, a standardized measure of the strength of sunburn-producing UV radiation, may partly contribute to this pattern, since UV-B irradiation of skin triggers vitamin D synthesis^2,3^. Meta-analyses suggest that serum levels of vitamin D are lower in patients with AA, supporting the potential role of vitamin D in the pathogenesis of AA^2^. However, only one included study was based on AA patients in the United States (US) suggesting lack of data in the US. Using UV index as an indirect surrogate of serum vitamin D levels, in the present study, we explore the association between AA flare frequency and various climate factors, including UV index.

## Methods

Medical charts of 457 children in Philadelphia, Pennsylvania with AA, alopecia totalis (AT) or alopecia universalis (AU) were reviewed. Of these, 336 children with dates of AA flare episodes in 2017 were included. Included episodes were the onset of hair loss, the first flare, and the second flare. Flares were defined as an increase in scalp hair loss noted by either patient or caregiver. Increased hair loss occurring immediately after a course of oral steroids was not included as a true flare episode to limit the confounding effect of steroids. Data from 518 episodes of flares were collected to calculate the frequency of flares during each month. The monthly values of climate variables in Philadelphia during 2015 to 2017 were obtained from World Weather Online, including average ambient temperature, UV index, air pressure, relative humidity, cloudiness, wind gust, wind speed, number of days with rain, volume of precipitation, number of days with sun, and hours of sunlight. Spearman rank correlation analyses were conducted using STATA version 15 to assess the correlations between these climate variables and AA flare frequency per month. To explore lag-time effects, UV index of the prior month was also analyzed.

## Results

Demographic information of the subjects was previously reported^1^. The male-to-female ratio was 1:1.5. The majority of our subjects self-identified as White (52.2%), followed by Black (19.6%), Hispanic or Latino (8.9%), Asian (4.1%), Indian (3.9%), Other (8.9%), and unknown (< 3%). The majority of our subjects had a documented atopic comorbidity (63.8%), with 87 cases of atopic dermatitis (AD), 47 allergic rhinitis, 35 asthma, 14 anaphylactic food allergy, and 2 eosinophilic esophagitis.

**Figure 1:**
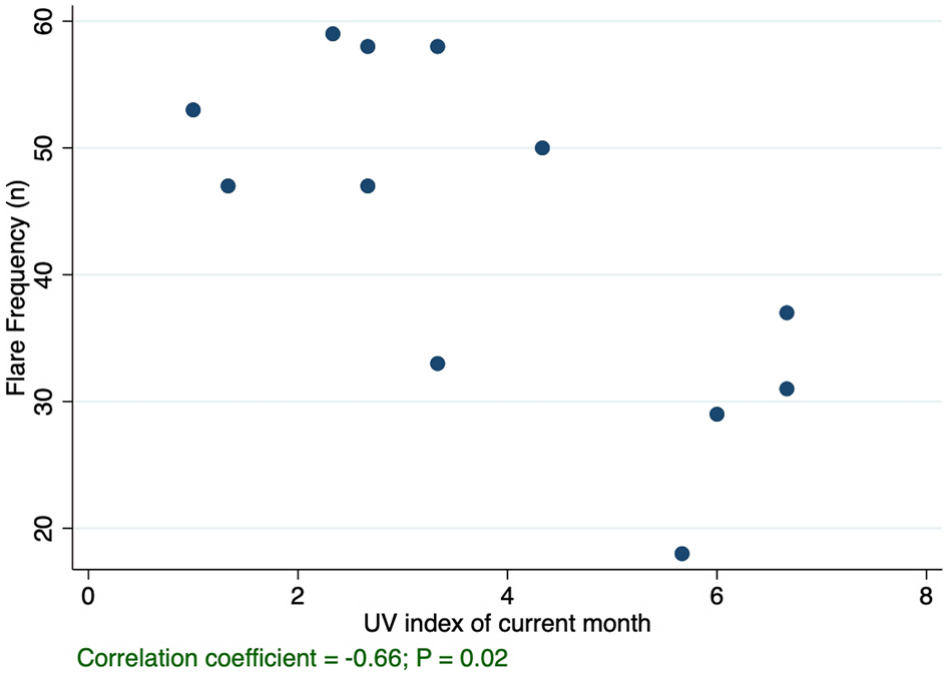
Scatterplot of alopecia areata (AA) flare frequency by UV index per month for children with AA in Philadelphia, Pennsylvania, US.

There were significant (P < 0.05) correlations between flare frequency and UV index (R = − 0.66), precipitation (R = − 0.66), number of days with rain (R = − 0.70), number of days with sun (R = 0.62), and air pressure (R = 0.80). (Table 1 and Fig. 1). Stratified analyses based on the presence of an atopic comorbidity revealed that the correlations between AA flares and certain climate variables, namely UV index and precipitation, were stronger and significant only in atopic patients with AA (Table 1). In atopic AA patients, new significant correlations appeared with temperature (R = − 0.75), wind speed (R = 0.64), and UV index of the prior month (R = − 0.61). However, in patients who did not have atopic comorbidities, we generally observed weaker and non-significant correlations between climate and AA flare frequency. Out of the twelve examined climate variables, only three remained significant in patients without atopic comorbidities: number of days with rain (R = − 0.63), number of days with sun (R = 0.58), and air pressure (R = 0.59).

**Table 1:** Correlation coefficients (Rs) between the frequency of alopecia areata (AA) flares and meteorological variables per month in Philadelphia, Pennsylvania, for children with AA, for children with comorbid AA and atopic conditions, and for children with AA without atopic conditions.

	All AA		AA with atopy		AA with no atopy	
	R	*P* value	R	*P* value	R	*P* value
Air pressure (mb)	0.80	0.002	0.79	0.002	0.59	0.04
Number of rainy days	− 0.70	0.01	− 0.63	0.03	− 0.63	0.02
UV index: current month	− 0.66	0.02	− 0.81	0.001	− 0.38	0.23
Precipitation (mm)	− 0.66	0.02	− 0.75	0.005	− 0.43	0.17
Number of sunny days	0.62	0.03	0.55	0.06	0.58	0.05
Temperature (celsius)	− 0.54	0.07	− 0.75	0.005	− 0.27	0.40
Wind speed (km/h)	0.45	0.14	0.64	0.02	0.16	0.62
UV index: prior month	− 0.33	0.30	− 0.61	0.03	− 0.053	0.87
Wind gust (km/h)	0.28	0.38	0.55	0.07	< 0.01	> 0.99
Hours of sunlight	− 0.28	0.38	− 0.55	0.07	< 0.01	> 0.99
Cloudiness (%)	0.20	0.53	0.45	0.15	0.07	0.83
Humidity (%)	− 0.18	0.59	0.03	0.93	− 0.30	0.35

Number of flare episodes in atopic AA patients: 198.

Number of flare episodes in non-atopic AA patients: 320.

## Discussion

As predicted, we found a strong inverse correlation between UV index and AA flare frequency, which may partly explain the seasonal pattern of AA that we previously observed^1^. Furthermore, this correlation was stronger among atopic AA patients compared with patients without atopy.

Our findings regarding UV index may support the potential role of vitamin D in AA pathogenesis as vitamin D deficiency is more common in the fall and winter months when UV exposure is lower^2,4^. UV-induced immunosuppression may contribute to these findings as well^5^. We also found significant correlations between flare frequency and precipitation, number of days with rain, and air pressure. Since these three climate factors correlated with UV index during the 3-year period (R = − 0.80 to 0.65), UV index may partly account for their significant association with AA flare frequency. While UV index was strongly correlated with temperature (R = 0.92) and UV index of the prior month (R = 0.82), associations between these climate variables and flare frequency were only significant in atopic AA patients.

Stratified analyses based on comorbid atopy suggest that atopic AA patients may be more susceptible to the influence of climate compared to those with no history of atopy (Table 1). This may be related to the fact that atopic conditions are associated with climate. Prior studies have found convincing evidence that atopic conditions have seasonal patterns and may be influenced by climate^6–9^. Several population studies based in the US^10^, Turkey^11^, Denmark^8^, and Norway^12^, but not others^7,13^, have suggested that there is an inverse relationship between temperature and AD. Lower temperatures correlated with increased AD prevalence^10^, more office visits^8,11,12^, more hospitalizations^8^, and more AD prescription medication utilization^8^. Prior studies have also found that some climate factors such as precipitation^10^, air pressure^11,14^, and UV index^15–17^, which were also the factors associated with AA in our study, were associated with AD. A meta-analysis based on nine individual studies found that AD was significantly associated with birth during the fall and winter seasons, compared with birth during spring^18^. One theory to explain the effects of season of birth on atopic conditions involves vitamin D produced by skin in response to UV-B radiation^19^. A recent systematic review supported the role of vitamin D in the pathogenesis of AD^20^.

Our study was based in a geographic region with four distinct seasons, which provides a wide range of climate variation. Our focus on a pediatric population may be advantageous, as high-dose supplemental vitamin D intake is less prevalent among children compared to adults^21^. This would allow for more robust influence of UV exposure on flare frequency, if serum vitamin D levels truly mediate the correlation between UV index and AA flare frequency. However, we were not able to explore the relationship between atopic severity and that of AA due to lack of information on atopic severity, which is one limitation of this study. Previous research indicates that allergy may contribute to AA pathogenesis in a subset of patients^22^. In fact, atopic AA patients may benefit from antihistamines as adjunct therapy to support hair regrowth^23,24^. Thus, it is reasonable to wonder whether antihistamine use and atopy severity contribute to the seasonal AA flare pattern that is observed in atopic AA patients. Future research studies including multivariable analyses may be useful to further define the relationship between climate, atopic flares, and those of AA.

In conclusion, UV exposure and other climate factors may be partially responsible for the seasonal pattern of AA flares and may contribute to AA pathogenesis. Physicians may counsel pediatric patients and their caregivers on these relationships between climate and AA flare frequency. A useful teaching analogy may be to think of the hair as leaves, with increased shedding during fall and more growth during spring. The seasonal patterns of AA flares should be documented in adults and pediatric populations in various geographic regions to better understand of the mechanisms.

## Data Availability

Dr. Cho and Ms. George had full access to all the data in the study and takes responsibility for the integrity of the data and the accuracy of the data analysis.
